# Evaluating the cost and operational context for national human papillomavirus (HPV) vaccine delivery in three regions of Ethiopia

**DOI:** 10.1371/journal.pgph.0003357

**Published:** 2024-07-15

**Authors:** Mercy Mvundura, Amare Bayeh, Meseret Zelalem, Yohannes Lakew, Adugna Dhufera, Belayneh Dagnew, Rose Slavkovsky, D. Scott Lamontagne

**Affiliations:** 1 Medical Devices and Health Technologies, PATH, Seattle, Washington, United States of America; 2 Ethiopia Country Office, PATH, Addis Ababa, Ethiopia; 3 Maternal and Child Health, Federal Ministry of Health, Addis Ababa, Ethiopia; 4 Ethiopia Public Health Institute, Addis Ababa, Ethiopia; 5 Center for Vaccine Innovation and Access, PATH, Seattle, Washington, United States of America; 6 JSI Research & Training Institute, Arlington, Virginia, United States of America; 7 Formerly Center for Vaccine Innovation and Access, PATH, Seattle, Washington, United States of America; PLOS: Public Library of Science, UNITED STATES OF AMERICA

## Abstract

Cervical cancer is the second leading cause of cancer deaths among women in Ethiopia. Human papillomavirus (HPV) vaccination is a primary prevention method for cervical cancer and was introduced in Ethiopia in 2018. We conducted a cross-sectional, mixed-methods study to understand the operational context for the HPV vaccination program and the associated costs in three regions of Ethiopia. Operations research provided insights on the frequency and intensity with which HPV vaccination program activities were done, focusing on activities conducted to vaccinate the cohort receiving its first dose in October 2019 and second dose in January 2021. Microcosting was used to estimate the costs to the health system for these activities. Data collection using structured questionnaires was done at 60 health facilities, 17 woredas, and 9 zones/sub-cities that were randomly selected from three purposively selected regions and the national level. Financial costs (monetary outlays) and economic costs (financial costs plus opportunity costs of resource use) were estimated in 2019 US$. Health facilities delivered an average of 411 HPV vaccine doses during the reference period, ranging from a mean of 86 to 606 across the three regions. Aggregated across all levels of the health system, the estimated financial cost per dose across the pooled sample was $2.23, and the economic cost per dose was $7.19, excluding the cost of vaccines and supplies. There were regional variations in these estimates, with mean financial cost per dose ranging from $1.17 to $7.18 and mean economic cost per dose ranging from $5.80 to $18.13 across the three regions. Regional variations exist in the service volume, frequency, and intensity of conducting HPV vaccination activities, as reflected in the estimated costs of delivery. Data generated from this study can be used to inform program planning and budgeting, taking into account regional variations, for effective utilization of resources.

## Introduction

In Ethiopia, cervical cancer is the second most frequent cancer and the second leading cause of cancer deaths among women [1]. The cervical cancer incidence rate was estimated as 21.5 cases per 100,000 women in 2020, with approximately 7,445 new cases and 5,338 deaths each year from the disease [1]. Globally, there are an estimated 604,127 cervical cancer cases and 341,831 deaths annually. Ethiopia ranks sixteen in the number of cervical cancer cases and eleventh in cervical cancer deaths [1].

Human papillomavirus (HPV) vaccines, as primary prevention of cervical cancer, are recommended by the World Health Organization (WHO) for inclusion in all national immunization programs [2]. With Gavi funding support, Ethiopia introduced HPV vaccine into their Expanded Programme on Immunization (EPI) in December 2018, targeting 14-year-old girls. In Ethiopia, HPV vaccines are administered primarily at schools; health facilities and non-school-based outreach at the community level are used in some regions to reach girls who may have been missed during school-based vaccination. Until July 2023, the immunization schedule in Ethiopia was two doses with at least a six-month interval between doses. In 2019, the administrative uptake rate for the first dose of HPV vaccine was 91% and the two-dose coverage rate was 82%; the two-dose coverage rate dropped to 74% in 2022 [3]. Since August 2023, Ethiopia shifted to single-dose HPV vaccination following the WHO Strategic Advisory Group of Experts on Immunization (SAGE) recommendation given the comparable efficacy and duration of protection as the two-dose schedule as well as the programmatic advantages [4].

Ethiopia has an estimated population of more than 120 million people [5]. The country is divided into 12 regions, categorized as agrarian, pastoral, and urban contexts. The immunization program in Ethiopia is coordinated from the national to the lowest community level through the Federal Ministry of Health, regional health bureaus, zones/sub-cities’ health offices, woreda (district) health offices, and primary health care units. Vaccination and other promotive and preventive health services are provided at the primary health care level, which is made up of health centers and health posts; hospitals also provide vaccination services, but their contribution is small. Health posts provide limited curative services, while health centers provide a broader range of primary health care services. In our analysis we use the term health facilities to jointly refer to health posts and health centers. The national coverage rate for routine infant vaccination is 61% for the third dose of pentavalent vaccine with regional variations ranging from 83% in Addis Ababa to 19% in Somali region [6]. This is due to context differences including infrastructure, health system challenges, and vaccine reach in pastoral versus urban communities. Across the regions, immunization coverage tends to be lower in regions such as Afar, Gambella, Benishangul-Gumuz, Somali, Tigray, and Amhara because of conflict, climate change (drought and flooding), remoteness, limited infrastructure, and other health emergencies.

There is limited evidence on the operational context and costs for routinely implemented HPV vaccine delivery in Ethiopia and other low- and middle-income countries, as most studies are conducted during vaccine demonstration programs or at introduction. Costing studies of HPV vaccine demonstration programs have limitations for national inference due in part to small sample sizes and a focus on measuring introduction costs, which impacts their accuracy and generalizability. After the initial introduction into the national immunization program, capturing new information on program operations and costs after the program becomes more routine can be useful for planning, budgeting, and decision-making for sustainability. Further, a more nuanced look at how program context differs between regions would help program managers better understand implementation and regional variations in costs and factors contributing to these costs.

Our study sought to address the limitations of prior research by estimating the ongoing delivery costs of the national HPV vaccination program and exploring the operational context for the two-dose HPV vaccination schedule. Specifically, our objective was to estimate the ongoing financial and economic costs and operational context of HPV vaccine delivery in three regions of Ethiopia (Addis Ababa, Afar, and Amhara).

## Materials and methods

### Study design

The study design has been previously described and the Ethiopia study was included as part of a six- country study [7]. In brief, this was a cross-sectional, mixed methods study that included two embedded research components: (1) operational research to quantify and describe HPV vaccination program implementation, assessing what was done, how, how often, and by whom; and (2) microcosting to identify, measure, and value the resources used for HPV vaccination program activities, using an ingredients-based methodology. For both the operational research and microcosting, we evaluated several HPV vaccination program activities including vaccine procurement; program planning and management; social mobilization and information, education, and communication (IEC) materials development and distribution; training; vaccine collection or distribution and storage; service delivery; supervision; record keeping; waste management; and crisis management.

For the costing evaluation, both financial costs and economic costs borne by the health system were estimated with no tracking of the payor. “Financial costing” is defined as accounting for transactions where there are monetary expenditures. “Opportunity costs” is defined as the costs of using existing resources, such as capital equipment, human resources, etc., even when a direct financial outlay is not incurred by the HPV vaccination program. The sum of the financial and opportunity costs is the “economic cost” [8]. The types of financial costs we accounted for as part of HPV vaccination program activities included per diems paid; meeting costs including venue rentals and meals; costs for vehicle rental and payment for public transport; expenditures for fuel for vehicles and other equipment, energy for cold chain equipment, and vehicle maintenance; and radio messages or printing and distribution of materials for social mobilization. The types of opportunity costs included for HPV vaccination program activities were health worker time; non-health worker (Ministry of Education staff, community stakeholders, and volunteers) time; and annualized costs for vehicles and equipment (incinerators, refrigerators, and vaccine carriers).

This study focused on evaluating the context and costs for HPV vaccine delivery for the cohort of girls receiving their first HPV vaccine dose in October 2019 and second dose in January 2021, in addition to the next annual cohort of 14-year-old girls receiving their first dose at this time. For each time period, vaccination activities were conducted over a 5-day period. The typical two-dose dose schedule timing was interrupted and extended due to system disruptions caused by the COVID-19 pandemic.

### Sample selection and sample size

Sample selection was done through a multi-step process. First, a purposive, criteria-driven selection of regions was done in consultation with the Federal Ministry of Health to represent urban, pastoral, and agrarian areas of the country. Addis Ababa (urban), Afar (pastoral), and Amhara (agrarian) regions were selected. The HPV vaccine target population in 2021 was approximately 21,300 for Addis; 13,400 for Afar; and 276,000 for Amhara, as obtained from secondary administrative data in the health information system in Ethiopia. In the second step, stratified random sampling, proportional to population size, was done using the Sample Design Optimizer tool [9] to select zones or sub-city health offices and woredas in each of these selected regions. In the third step, the health facilities (health centers or health posts) affiliated with the subnational administrative levels were selected using simple random sampling.

At the direction of the government of Ethiopia, zones, sub-cities, or woredas with security concerns were excluded from the sampling frame. Data were also collected at the national level. The sample size in each study region is shown in Table 1.

**Table 1:** Study sample sizes by region.

	Addis Ababa	Afar	Amhara	Total in sample
**National level**	Not applicable	Not applicable	Not applicable	**1**
**Regional health bureau**	1	1	1	**3**
**Zones or sub-cities**	5	Not applicable	4	**9**
**Woreda health offices**	Not applicable	5	12	**17**
**Health facilities (health centers and health posts)**	15	10	35	**60**

### Data collection

Data were collected from March 29 to April 17, 2022, using a structured interview questionnaire for operations and costs. The questionnaire was administered to health facility staff involved in HPV vaccine delivery activities at the selected study sites. Health facility staff provided verbal consent to participate in the study. Vaccination data were extracted from health facility records to capture location and dates of HPV vaccination sessions, number of vaccine doses used, and number of dose 1 and dose 2 vaccinations administered, depending on the available records. At the woreda, sub-city or zone, regional, and national levels, EPI focal points who manage the HPV vaccination program were interviewed as well with a questionnaire adapted for the administrative-level functions. All data were recorded electronically on computer tablets programmed with Open Data Kit software.

Secondary data (such as salary scales for staff working in the health and education sectors and replacement prices for equipment and vehicles) were collected from government documents and used as inputs in the costing analysis. In addition, quantities of HPV vaccine doses and other EPI vaccines used at each administrative facility were obtained from the health information system and used to estimate the allocation factors for shared resources, accounting for the share of HPV vaccines among other EPI vaccines.

### Data analysis

The operational context data were analyzed using software such as Excel (Microsoft Corporation, Redmond, Washington, USA) and SPSS (IBM Corporation, Armonk, New York, USA) and tabulated to provide information on the HPV vaccine delivery location and context. Counts and means of the continuous variables were computed; for categorical variables, frequencies were tabulated. The extracted data on doses used at health facilities were also analyzed to estimate session frequency and administration setting using SAS Studio (SAS Institute Inc., Cary, North Carolina, USA) to provide information on doses delivered over the reference period by each health facility in the sample.

All cost-related data were collected in Ethiopian birr and converted to 2019 US$ using an exchange rate of 32 Ethiopian birr per US dollar, which was the average exchange rate during the reference period for the analysis. Costing data analysis was done using Stata (StataCorp LLC, College Station, Texas, USA). Table 2 shows the unit prices used for the costing analysis.

**Table 2:** Unit costs in 2019 US$.

	Unit prices in US$
**Energy**	
Petrol price per liter	$0.72
Diesel price per liter	$0.81
Kerosene price per liter	$0.89
**Equipment and vehicles**	
Four-wheel drive truck	$88,820
Constructing a new incinerator	$8,233
**Median or range of monthly salary for Ministry of Health staff**	
Health workers level 3 to level 16	$144 to $385
Cold chain officers/pharmacists	$283
**Median or range of monthly salary for non-health workers**	
Teachers	$193
Other school staff	$78
Ministry of Education technical officers	$312 to $347
**Volume-based proportions used for allocating shared resources (ranges by region)**	
Addis	0.028 to 0.074
Afar	0.092 to 0.16
Amhara	0.079 to 0.227

Annualized costs for capital were calculated assuming 10-year economic life for vehicles and cold chain equipment, with economic life discounted at a 3% rate. Since cold chain equipment used for storing HPV vaccines was shared with other EPI vaccines, costs were allocated based on proportional volume of HPV vaccines relative to other EPI vaccines, taking into account cold chain volume per dose and the quantities of vaccines stored at each facility per year. As vehicles were used for EPI and other activities including primary health care, the same proportional volume was applied and then further allocated based on the number of days spent on EPI relative to other shared uses, as reported by the respondents at data collection.

For human resources, time spent on HPV vaccination program activities was reported by each staff member involved in HPV vaccine delivery. When HPV vaccination program activities were combined with other immunization program activities, these were allocated to the HPV vaccination program based on the reported proportion of time spent per month on HPV vaccination versus other activities. For non-health staff, the health facility staff involved in HPV vaccination activities who answered the survey reported the number of non-health staff involved in each activity and the duration of the activity. This information was used to estimate the opportunity costs of non-health worker time, using the relevant time valuation rates (Table 2).

Estimated costs are presented by level of the health system (health facility, woreda, zone/sub-city, region, and national level); by cost category (e.g., per diems, meeting costs, health worker time); by activity (e.g., vaccine procurement, training, service delivery); and also by regional subgroups. Costs exclude the value of vaccines and immunization supplies such as syringes. All costs represent the ongoing HPV vaccination program cost during the reference period of the study.

We report weighted mean costs per health facility or administrative office. We also calculated the volume weighted mean cost per dose using standard methodological guidelines [9], which was calculated as:

$$Volume\;weighted\;mean\;cost\;per\;dose=\frac{\sum_{i=1}^{n}{Cost}_{i}}{\sum_{i=1}^{n}{Doses}_{i}}$$

where *i* is each site in the study sample at that level of the health system and *n* is the sample size for that level of the health system.

### Ethics review

The study was approved by the Ethiopian Public Health Institute Institutional Review Board. The study was determined as not human subjects research by the PATH Research Determination Committee and so did not require oversight from a US-based ethics review committee. The national and regional immunization program managers provided administrative approval for immunization program staff in their jurisdiction to be interviewed and verbal consent was also obtained and documented by the research team from each of the immunization program staff at each study facility before the questionnaire was administered. Verbal consent was stated in the protocol that was approved by the ethics committee.

## Results

### HPV vaccine service delivery characteristics

Most health facilities sampled (51/60, 85%) conducted HPV vaccination activities during the study period. Nine facilities (15% of sample) reported supportive activities to health posts, rather than direct service provision. There were 214 HPV vaccination sessions conducted with approximately 21,000 doses administered by health facilities. The majority of HPV vaccination sessions conducted (89%) were at schools, 8% of sessions were routine outreach, and 3% were conducted at health facilities. Girls aged 14 years were eligible for HPV vaccination, and most HPV dose 1 vaccines were administered in this age group, with HPV dose 2 vaccines being split between girls aged 14 years and 15 years. In our sample, 62% of the doses delivered were given for the first dose of HPV vaccine. School-based delivery was considered routine outreach (i.e., no separate funds given) by 76% of the survey respondents and as special outreach (i.e., separate funds given) by 15% of the respondents.

**Fig 1:**
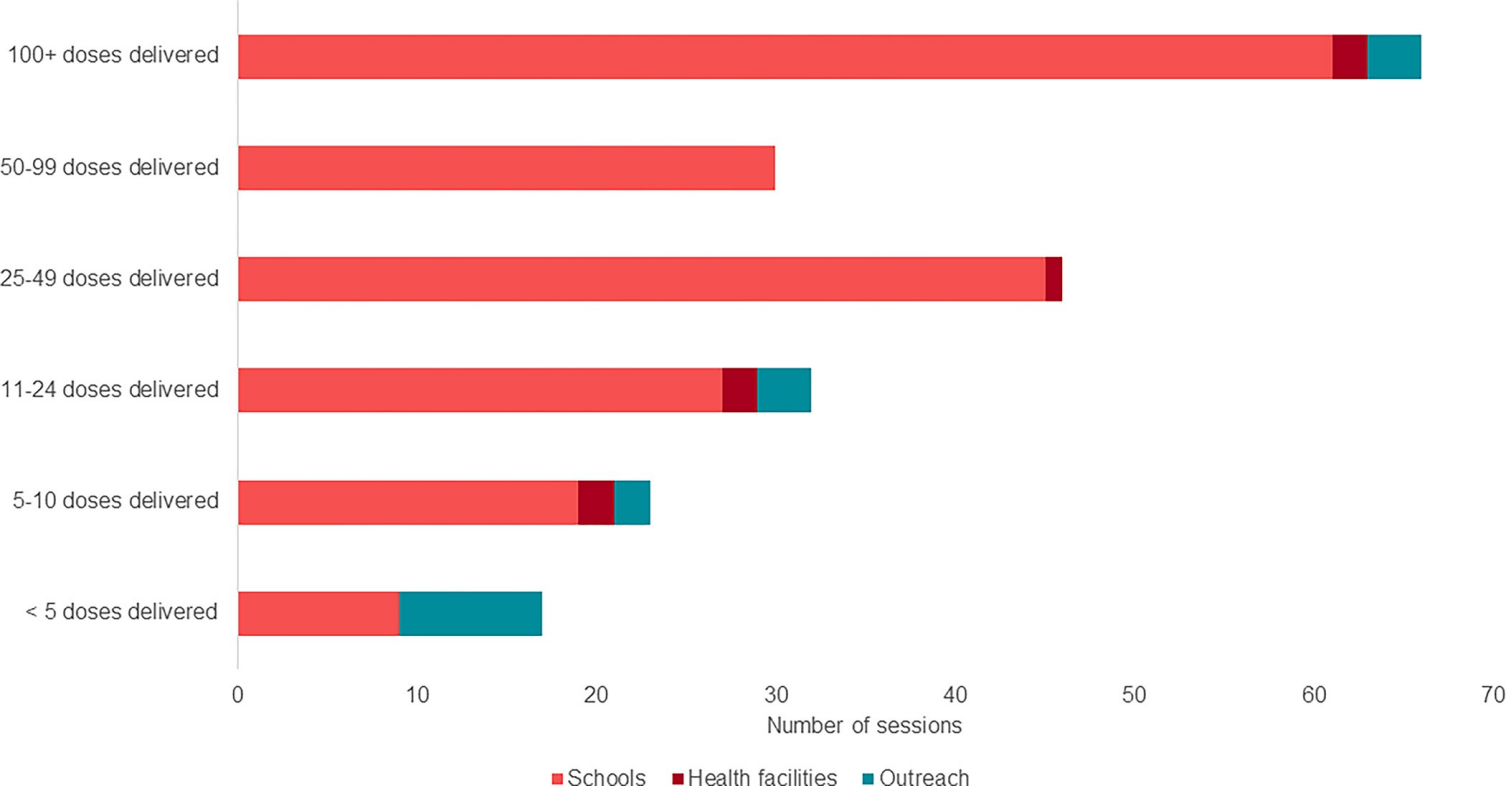
Total HPV vaccination session size, by service delivery location.

Vaccination sessions tended to be large whether conducted at schools, outreach or facility locations, averaging 93 to 118 doses administered per session (Fig 1). Sessions at schools were expected to capture more girls given the congregational aspect of a school setting. Outreach and facility-based HPV vaccination sessions disproportionately were skewed to smaller session sizes, even though one-third of sessions at these locations delivered more than 100 doses.

Operational context for HPV vaccine delivery at health facilities varied by region (Table 3). The mean number of doses administered by health facilities was lowest in Afar (86 doses), which is a pastoral region with sparser population. Across the study regions, there were also variations in the frequency and intensity with which HPV vaccination program activities were done. In Afar, a smaller proportion of health facilities typically conducted the activities, and when activities were done, they were done with less intensity compared to the pooled study sample and the other two regions.

**Table 3:** HPV vaccination program operational context at health facilities in the study sample during the reference period, for the pooled sample and by region.

Activity	Variable	Ethiopia pooled sample (n = 60) [7]	Addis (n = 15)	Afar (n = 10)	Amhara (n = 35)
**Service delivery**	Average number of HPV vaccine doses delivered per vaccinating health facility	411	606	86	378
**Program planning and management**	Number (%) of health facilities reporting conducting the activity	47 (78%)	11 (73%)	6 (60%)	30 (86%)
	Average number of activities conducted per health facility when the activity was done	3.2	2.3	1.8	3.8
**Social mobilization and IEC**	Number (%) of health facilities reporting conducting the activity	45 (75%)	11 (73%)	4 (40%)	30 (86%)
	Average number of activities conducted per health facility when the activity was done	5.4	8.2	1.0	4.2
**Training**	Number (%) of health facilities reporting conducting the activity	35 (58%)	9 (60%)	3 (30%)	23 (66%)
	Average number of activities conducted per health facility when the activity was done	3.4	2.6	3.3	3.8
**Crisis management and response**	Number (%) of health facilities reporting conducting the activity	19 (32%)	4 (27%)	2 (20%)	13 (37%)
	Average number of activities conducted per health facility when activity was done	1.3	1.5	1.0	1.3
**Vaccine collection or distribution and storage**	Number (%) of health facilities collecting vaccines from higher-level facilities	17 (28%)	5 (33%)	3 (30%)	9 (26%)
	Average number of trips made to collect HPV vaccines (and other vaccines, if combined trips) per health facility when the activity was done	2.3	2.6	2.7	2.0
	Number (%) of health facilities with refrigerators for storing HPV vaccines (and other vaccines)	43 (72%)	15 (100%)	10 (100%)	18 (51%)

Abbreviations: HPV: human papillomavirus; IEC: information, education, and communication materials.

There was less variation in program operations at subnational administrative levels, though relatively more program planning activities were done in Addis than in the other two regions, and more training activities were done in Afar (Table 4). There was wide variation in the number of HPV vaccine doses delivered by region, as a reflection of population size and density in these different areas of the country.

**Table 4:** HPV vaccine delivery operational context at subnational administrative-level offices in the study sample by region.

Activity	Variable	Ethiopia pooled sample (n = 29) [7]	Addis (n = 6)	Afar (n = 6)	Amhara (n = 17)
**Program planning and management**	Number (%) of administrative offices reporting conducting the activity	27 (93%)	6 (100%)	5 (83%)	16 (94%)
	Average number of activities conducted per administrative office when the activity was done	3.7	5.3	3.4	3.1
**Social mobilization and IEC**	Number (%) of administrative offices reporting conducting the activity	28 (97%)	6 (100%)	5 (83%)	17 (100%)
	Average number of activities conducted per administrative office when the activity was done	2.0	3.0	1.0	2.0
**Training**	Number (%) of administrative offices reporting conducting the activity	21 (72%)	5 (83%)	4 (67%)	12 (71%)
	Average number of activities conducted per administrative office when the activity was done	4.9	3.6	6.5	4.8
**Crisis management and response**	Number (%) of administrative offices reporting conducting the activity	10 (34%)	4 (67%)	2 (33%)	4 (24%)
	Average number of activities conducted per administrative office when the activity was done	1.6	2.0	1.0	2.0
**Supervision**	Number (%) of administrative offices conducting supervision visits	28 (97%)	6 (100%)	6 (100%)	16 (94%)
**Vaccine procurement**	Mean number of HPV vaccine doses delivered during the reference period (2019 vaccination cohort)	Woreda: 2,132	Woreda: NA	Woreda: 749	Woreda: 2,708
		Zone or sub-city: 26,356	Zone or sub-city: 4,295	Zone or sub-city: NA	Zone or sub-city: 53,933
		Region: 197,174	Region: 47,859	Region: 25,240	Region: 518,422

Abbreviations: HPV; human papillomavirus; IEC: information, education, and communication materials; NA, not applicable.

### Costs for HPV vaccination program activities at health facilities

The mean financial cost per health facility in the pooled sample was estimated at $421 (95% confidence interval [CI]: $0–$975) and the mean economic cost was $1,550 (95% CI: $722–$2,375) [7]. Stratified by region, the mean financial and economic costs, respectively, were $1,369 and $2,881 for Addis; $143 and $614 for Afar; and $125 and $1,228 for Amhara.

**Fig 2:**
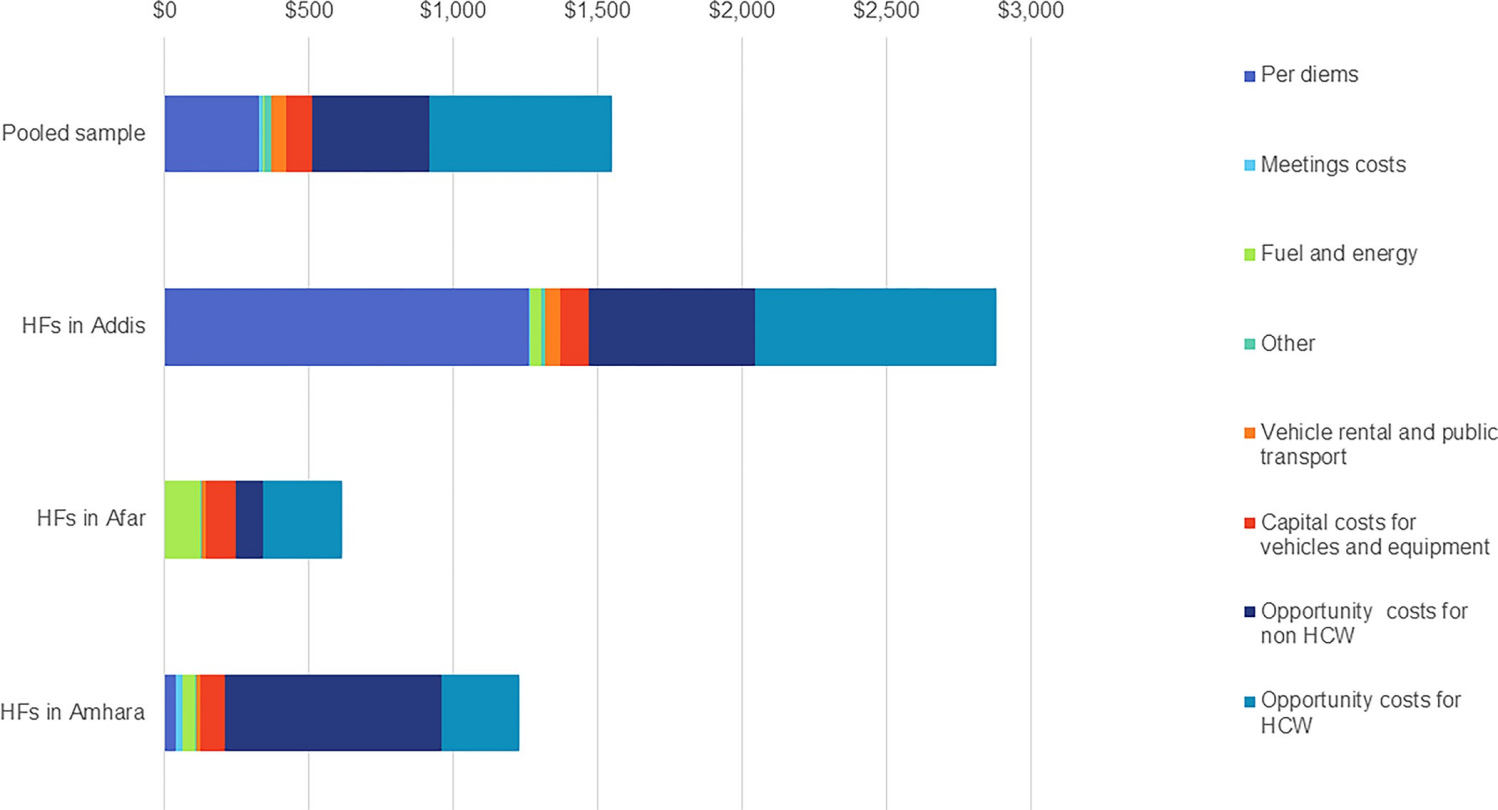
Mean economic costs by cost types at health facilities for the pooled sample and by region. Abbreviations: HCW, health care worker; HF, health facility.

Fig 2 shows the weighted mean economic costs for HPV vaccination program activities per health facility for the pooled sample and when the health facilities are in regional subgroups. Financial costs were 27% of the mean economic costs for the pooled sample of health facilities. These financial costs were mainly per diems paid for the HPV vaccination program activities, which accounted for 77% of the financial costs. By regional subgroups, financial costs were 48%, 23%, and 10% of mean economic costs for the health facilities in Addis, Afar, and Amhara regions, respectively. In Addis, per diems accounted for the largest share of financial costs (92%), and in Afar, fuel accounted for 85% of the financial costs. In Amhara, financial costs comprised mainly fuel costs (37%) and per diems (32%).

Across the pooled sample and for the regional subgroups, opportunity costs were the larger share of the economic costs, accounting for at least 52% of the economic costs. For the pooled sample and for the Afar and Amhara regions, at least 60% of the economic costs were opportunity costs of health worker and non-health worker time. In Addis, 49% of the economic costs were opportunity costs of this human resource time.

**Fig 3:**
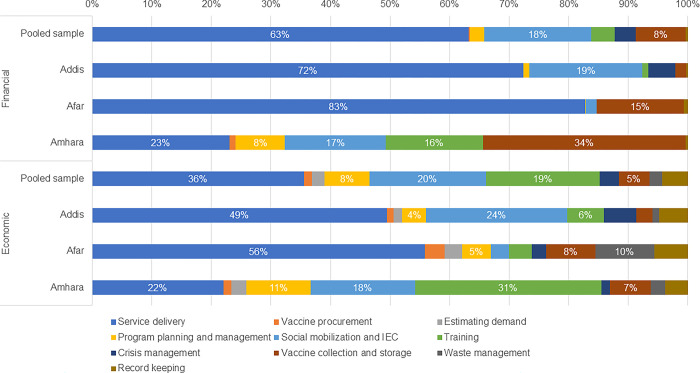
Proportion of financial and economic costs by activity at health facility level. Abbreviation: IEC, information, education, and communication.

The activities accounting for the largest share of costs at health facility level also varied by region (Fig 3). Financial costs at the health facility level were expended mostly (at least 63%) for service delivery in the pooled sample and also in Addis and Afar. However, for Amhara, financial costs were expended mainly for vaccine collection, with service delivery being the activity with the second largest share of financial costs. In Addis, service delivery costs accounted for 49% of the mean economic costs, followed by 24% for social mobilization. In Afar, service delivery accounted for 56% of the mean economic costs, with other activities each accounting for 10% or less of the costs. In Amhara, training accounted for 31% of the mean economic costs, with service delivery accounting for 22% and social mobilization for 18%.

### Costs for the HPV vaccination program at administrative levels

Table 5 shows financial and economic costs at the administrative levels. These are not reported disaggregated by region due to small sample sizes, especially for the upper subnational levels of the health system. Mean financial and economic costs at woreda level for the study sample were estimated at $678 and $4,833 [7], respectively, with financial costs accounting for 14% of the mean economic costs at this level. Financial costs were composed of fuel costs (43%) and per diems (39%). For the woredas in the sample, crisis management, training, and program planning and management were the activities that had the largest economic costs. The bulk of the costs for these activities were opportunity costs of time for non-health workers given the large numbers of school staff and community stakeholders reported to be engaged in these activities (not shown in table).

**Table 5:** Weighted mean financial and economic costs at administrative levels for HPV vaccination program activities in 2019 US$ and percentages of total costs.

Activity	Woredas (n = 17)		Zones and sub-cities (n = 9)		Regions (n = 3)		National level (N = 1)	
	Financial costs	Economic costs	Financial costs	Economic costs	Financial costs	Economic costs	Financial costs	Economic costs
Program planning and management	$140 (21%)	$1,031 (21%)	$7,291 (47%)	$7,453 (40%)	$7,651 (17%)	$7,822 (13%)	$16,188 (19%)	$16,188 (17%)
Social mobilization and IEC	$32 (5%)	$85 (2%)	$0 (0%)	$0 (0%)	$9,498 (21%)	$9,724 (17%)	$33,942 (39%)	$34,098 (35%)
Training	$9 (1%)	$1,194 (25%)	$5,736 (37%)	$7,656 (41%)	$22,422 (50%)	$31,653 (54%)	$0 (0%)	$0 (0%)
Crisis management	$9 (1%)	$1,331 (28%)	$9 (<1%)	$48 (<1%)	$0 (0%)	$0 (0%)	$19,592 (23%)	$21,202 (22%)
Vaccine collection or distribution and storage	$239 (35%)	$385 (8%)	$571 (4%)	$735 (4%)	$1,235 (3%)	$1,481 (3%)	$2,880 (3%)	$5,897 (6%)
Waste management	$65 (10%)	$94 (2%)	$0 (0%)	$0 (0%)	$0 (0%)	$17 (<1%)	$0 (0%)	$0 (0%)
Record keeping	$24 (3%)	$24 (<1%)	$30 (<1%)	$30 (<1%)	$0 (0%)	$0 (0%)	$0 (0%)	$0 (0%)
Supervision	$159 (24%)	$163 (3%)	$1,866 (12%)	$1,883 (10%)	$3,786 (8%)	$7,541 (13%)	$14,149 (16%)	$15,413 (16%)
Human resources	$0 (0%)	$526 (11%)	$0 (0%)	$680 (4%)	$0 (0%)	$366 (1%)	$0 (0%)	$4,042 (4%)
**Total costs** (weighted mean and 95% CI) [7]	$678 ($267–$1,088)	$4,833 ($273–$9,441)	$15,503 ($6,270–$37,277)	$18,485 ($3,427–$40,396)	$44,592	$58,604	$86,751	$96,840

Abbreviations: CI, confidence interval; IEC, information, education, and communication.

Note for woredas, zones and sub-cities, and regions, these are the means and ranges (as relevant) for the sample of facilities included in our study.

At the sample of sub-city and zonal health departments, financial costs accounted for 85% of the mean economic costs, which totaled $18,485. Financial costs mostly comprised expenses for hosting meetings (75%). Program planning and training were the two activities accounting for the largest share of costs at this level, with a large share of expenditures for meeting costs. Similar to the findings for the sub-cities and zonal health departments, financial costs were the largest share (76%) of costs at the regional health departments in our sample, which had a mean economic cost of $58,604. Financial costs comprised meeting costs (39%) and per diems (32%). Training was the activity with the greatest costs. Per diems accounted for 24% and meeting costs accounted for 26% of the economic costs at this level (not shown in table). The time costs for non-health workers to attend HPV vaccination program activities accounted for 23% of the economic costs.

At the national level, financial costs ($86,751) were the largest share (90%) of the economic costs ($96,840). Financial costs were mainly per diems (44%), IEC materials (25%), and meeting costs (22%). Social mobilization, crisis management, and program planning and management were the activities accounting for the largest cost shares. Per diems accounted for 39% of the economic costs and were the single largest share of costs, followed by costs for IEC materials (not shown in table).

### Aggregated costs across all levels of the health system

**Fig 4:**
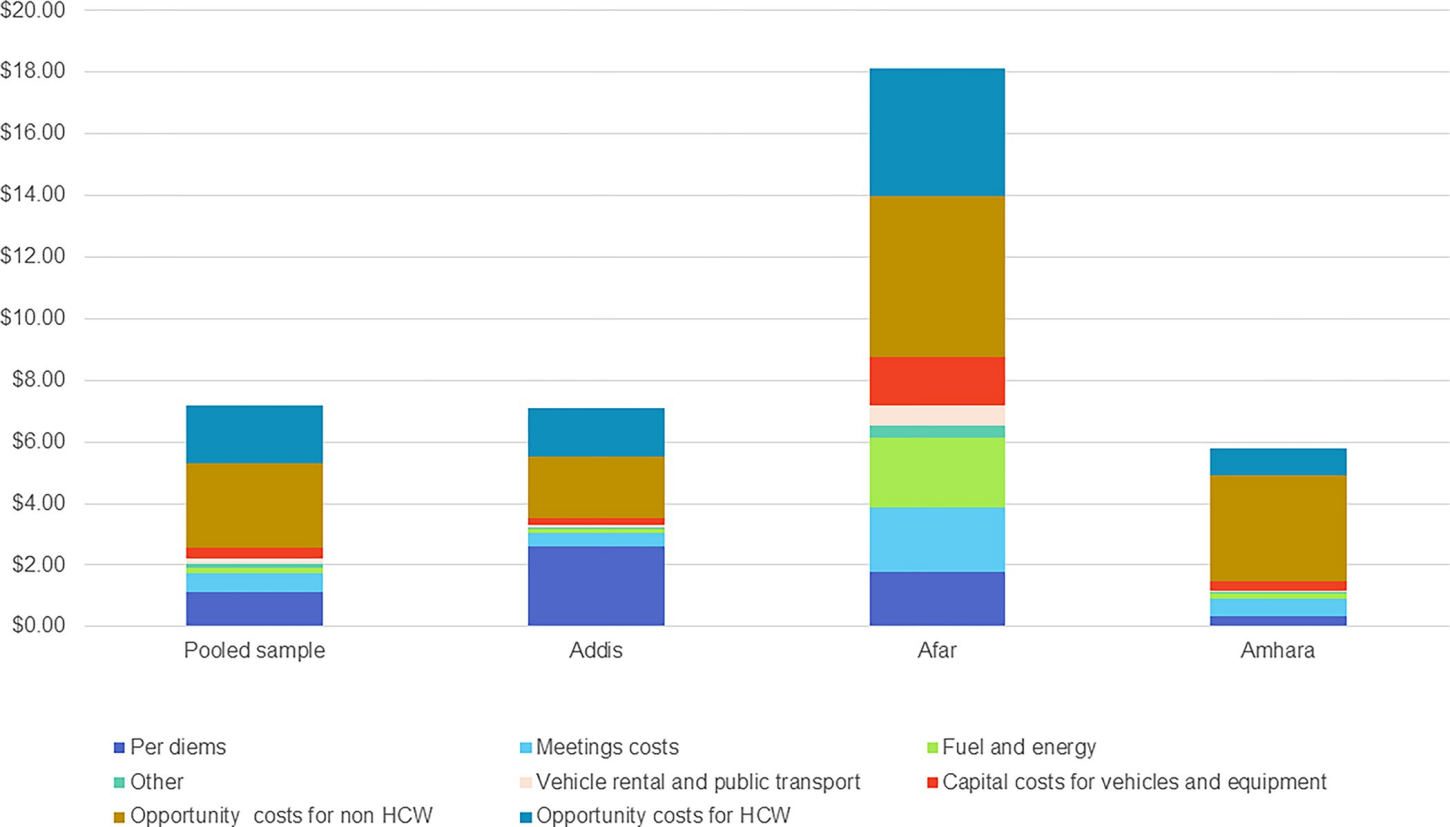
Mean economic costs per dose aggregated across all levels of the health system and by regional subgroups. Abbreviation: HCW, health care worker.

The mean financial and economic cost per dose varied by region, compared to the total pooled sample (Fig 4): $3.32 and $7.12 in Addis, $7.18 and $18.13 for Afar, and $1.17 and $5.80 for Amhara, respectively, compared to $2.23 and $7.19 for the pooled sample [7]. The Afar region’s costs were significantly higher than the pooled sample, whereas the other two regions were closer to those estimates. This difference is partly due to proportionately lower doses delivered in Afar region. For the pooled sample, mean financial costs were 31% of the mean economic costs per dose aggregated across all levels of the health system, but in the specific regions, they were 47% in Addis, 40% in Afar, and 20% in Amhara. Per diems accounted for 16% of the mean economic costs in the pooled sample, but in Addis they were 37%, which is much higher than the 10% and 6% cost share for Afar and Amhara, respectively. Overall, opportunity costs of human resource time were the largest share of the economic costs per dose, being 65% in the pooled sample and ranging from 51% to 75% in the regional subgroups analysis.

## Discussion

This study evaluated the program context and costs for delivery of HPV vaccines on a two-dose schedule in three regions of Ethiopia. The study found that the Addis, Afar, and Amhara regions concentrated their HPV vaccination efforts largely in schools—where 89% of sessions were held—during two fixed points in time, each having 5-days of vaccination activities. There were stark differences in the number of doses administered by region, in part due to differences in target population size. Our study found that there were regional variations in the frequency and intensity of HPV vaccination program activities, with these variations more pronounced at the health facility level. At subnational administrative levels, there was less variability as most offices conducted program planning, social mobilization, training, and supervision.

Our study also found that costs varied by region. Financial costs per dose aggregated across all levels of the health system were approximately six times higher in Afar region ($7.18) compared to Amhara ($1.17). The economic cost per dose was approximately three times higher in Afar ($18.13) than Amhara ($5.80). Although Afar had the highest cost per dose, its intensity and frequency of HPV vaccination program activities was relatively lower at the health facility level and represented the lowest absolute costs in our sample, implying that the higher cost per dose may be driven by the lower service volume. Given this, as a pastoral region with a smaller population size, it may be challenging for Afar to achieve the efficiencies with scale that can be reached by more populated regions. Although several studies have reported country-level variations in HPV vaccine delivery costs [10–13] few have reported regional variations. In addition to the difference in cost, we also found that the operational context varies by region, as previously mentioned.

Our study also found differences by regional subgroups on the activities where financial spending was happening and what the funds were spent on. At the health facility level, in two of the three regions (Addis and Afar), financial spending was mostly for service delivery, with the funds spent on per diems in Addis and fuel cost to travel to the non-facility-based vaccination sites in Afar. In Amhara, however, financial spending was mainly on fuel for vaccine collection, though service delivery accounted for the second largest share of financial spending (for fuel and per diems for service delivery). The finding that service delivery is where the financial spending is happening is not surprising given this is the core activity done by this level of the health system. Geographic context may explain the relatively higher expenditure on fuel in Afar. There were also differences in activities where financial spending was happening at the administrative levels and cost types where the spending was happening. These differences may reflect variations in program areas of focus by different levels of the health system.

Global stakeholders tend to use a fixed cost per child allocation when providing HPV vaccination program support to a country [14] without accounting for regional differences in costs and operational context. Yet, our study shows that the costs of delivering a dose of HPV vaccine differs starkly between regions. Planning, budgeting, and funding allocations should take into account these cost differences by geography, especially when large differences exist in the cost of providing HPV vaccination services to the target population.

Our study found that the costs to deliver HPV vaccines and conduct other program activities are largely composed of opportunity costs, with the share of financial costs being smaller. Opportunity costs of human resource time accounted for between half and three-quarters of the cost per dose. Thus, the HPV vaccination program activities are human resource intensive, more so with the engagement of school staff and community stakeholders whose collaboration is needed for the program’s success.

For the reference period evaluated in this study, two cohorts were vaccinated in January 2021, as the second HPV vaccination cohort in the country received its second HPV vaccine dose while the third cohort received its first dose. This type of vaccination strategy, which includes multiple cohorts, increases the number of doses delivered and reduces the cost per dose of HPV vaccine delivery. Vaccination strategies that include targeting a multi-age cohort could further reduce HPV vaccine costs of delivery. Research from high-income countries suggests that multi-age vaccination is cost-effective in younger cohorts [15]; however, costing studies and health economic evidence from low- and middle-income countries is needed to better understand the cost implications of single- versus multi-age cohorts in this context. Other strategies, such as moving to a single-dose schedule, could be another opportunity for cost efficiency.

This study has several limitations. Due to the COVID-19 pandemic, implementation of the second dose of HPV vaccine was done almost 18 months after the first dose was administered. It is therefore possible that some HPV vaccination program activities had to be repeated, which may have inflated intensity and costs compared to if vaccination was done at the scheduled interval of six months between doses. However, we were not able to evaluate the impact of this implementation delay on our study estimates. Second, our study included just three regions, which may not represent the entire country, though they do represent the geographical variation in the country. Also, in the Afar region, the sample size for health facilities was small, which could have biased the results. In addition, there were challenges with the availability of records, such as tally sheets, as these types of records are not routinely used in the program. As a result, some details related to HPV vaccination sessions could not be obtained, and we had to rely on information from monthly reports and were not able to do a more detailed analysis on session data. We also failed to obtain information on expenditures for customs clearance and handling costs for HPV vaccines, despite several attempts during data collection; as a result, our cost estimates may be underestimated, though the impact on the costs estimates is likely to be small. Also, we were not able to provide evidence on how costs differ by service delivery location, as 89% of the doses were provided in school-based settings, which precluded an adequate sample size to make comparisons by delivery location. This can be an area of investigation for future studies. Our analysis also did not disaggregate activities and related costs by time of dose administration, but rather collected and reported data agnostic of the timing of each dose delivery. Finally, due to lack of specific data on when each cost was incurred, we used an average exchange rate over the reference period and did not apply different exchange rates for different time periods.

In conclusion, our study evaluated the HPV vaccination program context and costs in three regions of Ethiopia that implemented a predominately school-based delivery strategy. We found regional variations in the frequency and intensity of HPV vaccination program activities that were carried out, especially at the health facility level. There were also regional variations in the number of doses delivered, the cost per dose estimates, and what comprised these costs. The evidence generated from this study can provide local stakeholders with regionally specific cost data to inform a more nuanced understanding of the ongoing costs of HPV vaccine delivery. Global stakeholders will benefit from being informed of the regional variations in costs, which should be accounted for when making funding allocation decisions.

## Supporting information

S1 Checklist Inclusivity in global research. (DOCX)
